# The Optimization of HIV Testing in Eastern Indonesia: Findings from the 2017 Indonesian Demographic and Health Survey

**DOI:** 10.3390/healthcare10030533

**Published:** 2022-03-14

**Authors:** Kusman Ibrahim, Hidayat Arifin, Siti Ulfah Rifa’atul Fitri, Yusshy Kurnia Herliani, Hasniatisari Harun, Agus Setiawan, Bih-O Lee

**Affiliations:** 1 Department of Medical and Surgical Nursing, Faculty of Nursing, Universitas Padjadjaran, Bandung 45363, Indonesia; k.ibrahim@unpad.ac.id (K.I.); siti.ulfah.rifaatul@unpad.ac.id (S.U.R.F.); yusshy.kurnia@unpad.ac.id (Y.K.H.); hasniatisari.harun@unpad.ac.id (H.H.); 2 Department of Community Health Nursing, Faculty of Nursing, Universitas Indonesia, Depok 16424, Indonesia; a-setiawan@ui.ac.id; 3 College of Nursing, Kaohsiung Medical University, Kaohsiung 807, Taiwan; biholee@kmu.edu.tw

**Keywords:** HIV, public health services, Indonesia, rural population

## Abstract

There is a concerning increase in human immunodeficiency virus (HIV) incidence in eastern Indonesia. HIV testing rates in this area are the lowest in the country. This study aimed to analyze the determinants of HIV testing in the Public Health Centers (PHCs) in eastern Indonesia. A cross-sectional study design was utilized using secondary data from the 2017 Indonesian Demographic and Health Survey (IDHS). We focused the survey on eastern Indonesia (Sulawesi, Maluku, and Papua) with PHC settings. After we weighted and removed missing data, we obtained 2118 surveys (425 males and 1693 females aged 14–54 years). Chi-square and binary logistic regression were used to analyze the determinants of HIV testing optimization in eastern Indonesia with a significance level of *p* < 0.05. The HIV testing coverage at the PHCs in eastern Indonesia was found to be 28.28%. From the survey, we found that respondents from the province of Papua (AOR: 1.45; 95% CI: 1.09–1.91), those who were female (AOR: 2.37; 95% CI: 1.75–3.12), and those with more information on HIV (AOR: 1.88; 95% CI: 1.41–2.51) were more likely to undergo HIV testing at the PHCs. Meanwhile, the wealthiest respondents (AOR: 0.61; 95% CI: 0.42–0.89) and the respondents who engaged in the perpetuation of stigmatization (AOR: 0.65; 95% CI: 0.42–1.02) were less likely to undergo HIV testing at the PHCs. The coverage of HIV testing in eastern Indonesia needs special attention from the Indonesian government. Increasing equity, distributing information regarding HIV and acquired immunodeficiency syndrome (AIDS) through social media, and creating accessible HIV testing in rural areas are vital for developing appropriate interventions.

## 1. Introduction

The incidence of HIV is growing steadily in Indonesia, with over 670,000 HIV-infected individuals recorded in 2015 [1]. The United States Agency for International Development (USAID) and the Indonesian Ministry of Health have collaborated to achieve the Sustainable Development Goals (SDGs) target of eliminating HIV in 2030. The program of HIV testing and prevention promoted by USAID included 95% of people living with HIV (PLHIV) knowing their HIV status, 95% knowing the treatment, and 95% treatment with suppressed viral loads [2,3]. The Joint United Nations Programme on HIV/AIDS (UNAIDS) has a relevant program with SDGs for ending HIV/AIDS that aims to end poverty, end hunger, ensure healthy lives, ensure quality education, achieve gender equality, reduce inequality, and promote peaceful and inclusive societies among PLHIV [4].

Based on the Indonesian Health Profile data [5], the number of HIV-positive reports in adults remains high. We compared this with the data in 2018 when there were 50,282 HIV-positive cases recorded in Indonesia. Conversely, the number of new cases diagnosed with AIDS decreased to 7036 cases in 2019 compared to the average of 8000 in the prior years. The most afflicted include areas in eastern Indonesia, such as the province of Papua, where the transmission rate was 15 times greater than the national average in 2014 [5,6]. Papua is Indonesia’s easternmost province. High degrees of mobility have contributed to the spread of HIV, now assumed to be transmitted mostly through heterosexual encounters. Although Papua makes up just over 1% of Indonesia’s population, 30% of Indonesia’s official HIV/AIDS cases occur there [7].

Eastern Indonesia is an area that is far from the capital city of Indonesia, and this hampers the coverage of HIV testing and information dissemination due to cultural, socioeconomic, and demographic factors [8]. Efforts to improve the HIV testing coverage and to discover new cases of HIV infection are ongoing. Strategies to improve the HIV testing coverage have involved enabling the PHCs to provide HIV testing services and counseling as well as mobile HIV testing [9]. The Ministry of Health of the Republic of Indonesia with regulation No. 75 of 2014 states that PHCs are health service facilities that carry out public health efforts by prioritizing promotive and preventive actions to achieve the highest public health status in their working area [10]. PHCs were the first type of health facility established by the government to enhance comprehensive HIV testing. However, there is a need for special attention to be given concerning the regions in eastern Indonesia. The optimization of HIV testing in the PHCs is likely to reduce the incidence of patients with AIDS and prevent deaths [11]. However, previous studies have shown that there is still a lack of diagnostic possibilities in primary care. HIV testing services in the PHCs are still not integrated with clinical services. Furthermore, there is a limited ability of health workers to analyze the problem and plan an intervention effectively [11,12,13,14,15].

The limited number of statistics reporting HIV tests in the regions of eastern Indonesia indicates that the level of health of the Indonesian people is still varied [16]. This indicates the gap in the utilization of health services, such as PHCs, between urban and rural areas. In line with the previous studies, a further barrier to accessing HIV-related care was revealed, as it is dependent on the facility and social support available [17,18,19,20]. Eastern Indonesia has more rural demographic characteristics, as well as restricted transportation, information sources, and economic growth [21,22,23]. PHCs, as the community’s initial entrance to HIV prevention and treatment at the level of basic services to prevent a growth in the number of cases of HIV, should be reachable and accessed by all communities.

Due to the social, demographic, and economic characteristics, the number of studies on HIV testing in eastern Indonesia are minimal. We present data from the areas of Sulawesi, Maluku, and Papua in particular. This will provide an overview of eastern Indonesia, touching on various aspects including the region, age, gender, wealth index, level of education, place of residence, occupation of the respondents, income of the respondents, stigma, HIV-related knowledge, and HIV-related information. This study aimed to determine the associated factors related to the optimization of HIV testing in the PHC setting in eastern Indonesia.

## 2. Materials and Methods

### 2.1. Study Design

A secondary, cross-sectional analysis focusing on eastern Indonesia (Sulawesi, Maluku, and Papua) was conducted on the Indonesian Demographic Health Survey (IDHS) data of 2017.

### 2.2. Data Sources and Sample

The survey was conducted in December 2017. The 2017 IDHS was nationally conducted by Statistics Indonesia, in collaboration with national agencies such as the National Population and Family Planning Board and the Ministry of Health of Indonesia. This survey was funded by the Indonesian government and took place from July 24 to 30 September 2017, in 34 provinces. The survey was technically assisted by Inner-City Fund (ICF) International, through the Demographic and Health Surveys (DHS) Program [24]. The dataset was obtained from the DHS Program’s website (URL: https://www.dhsprogram.com/data/available-datasets.cfm. Accessed on 19 July 2021) by applying directly, and it was downloaded after approval was obtained.

The DHS Individual Recode and Male Recode were used in this study to obtain male and female surveys. DHS used the multistage cluster sampling design to provide the representative estimation information for all enumeration areas. DHS used probability proportional to size (PPS) methodologies in the designated locations in both rural and urban residences. Households were selected randomly in the enumeration areas that had been previously established and determined. All surveys included a weighted sample in the data collection. Weighting is a correction technique that is used in the survey study in order to improve the accuracy of the survey estimates [25]. We weighted the DHS data because the overall probability of selection of each household was not a constant [26]. To minimize the number of errors made when obtaining the desired information and to maximize validity and reliability, DHS provided policies for the use of the questionnaires, which were translated and printed in all of the major local languages in which the interviews were expected to take place [27].

**Figure 1:**
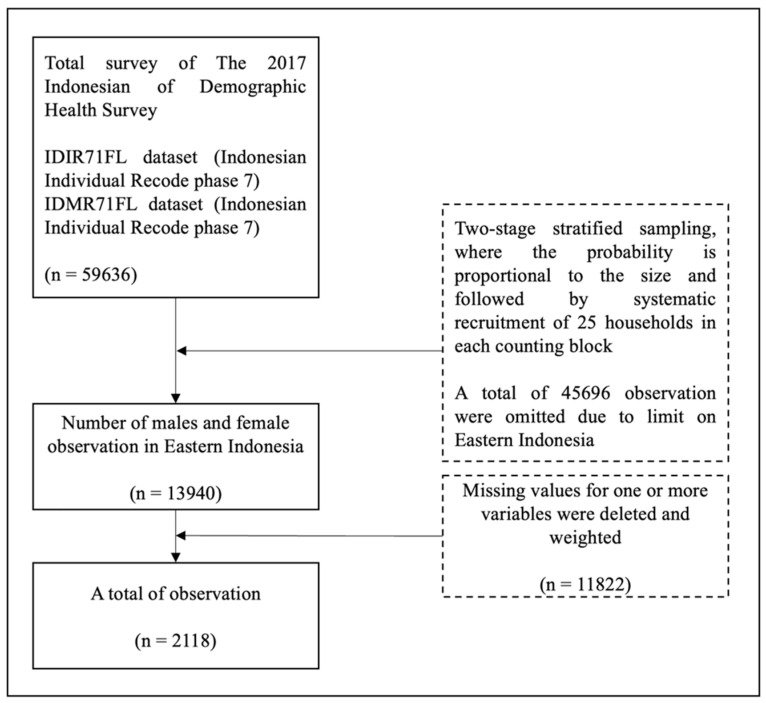
Flow Chart for Sample Size Selection.

A total of 59,636 responses were obtained from the survey. We focused the survey on eastern Indonesia with PHC settings. A total of 13,940 responses were specifically from eastern Indonesia. Surveys with missing data were eliminated, and a subset of 2118 was finally selected after the data were weighted. The inclusion criteria in this study were respondents aged 14–54 years. Any missing data were excluded from the study (Figure 1).

### 2.3. Variables

An HIV test is defined as an examination carried out as early as possible to prevent the transmission and decrease the incidence of HIV infection [28]. Respondents were asked “Have you ever HIV tested in a public health center?”, to which they could respond “Yes” or “No”.

The dependent variables in this study included region, age, gender, wealth index, level of education, residence, whether the respondents were working, the respondents’ earnings, stigma, HIV-related knowledge, and HIV-related information.

Eastern Indonesia was classified into Sulawesi, Maluku, and Papua based on the distribution of regional zones in Indonesia. We classified age into 50–54 years, 35–49 years, 25–34 years, and 15–24 years [29]. Gender was categorized into male and female. The wealth index was categorized into the richest, richer, middle, poorer, and poorest [30,31], which was determined based on principal component analysis (PCA) [32]. Education was categorized into high, secondary, primary, and no education [33]. Furthermore, the respondent’s place of residence was categorized into rural and urban [34]. The employment status of the respondent was determined based on their status of currently working (yes or no) and their earnings (paid or unpaid) at the time of the interview [35].

The stigma variable was defined as the respondents who engaged in and/or experienced stigmatization as it relates to people living with HIV (PLHIV). This variable was constructed from four questions that indicate stigma behavior, namely, “Would you want HIV infection in the family to remain secret?”, “Would you be ashamed if someone in the family had HIV?”, “Would you buy vegetables from a vendor with HIV?”, and “People talk badly about people with HIV or believed to have HIV?” [34]. We then recoded the variable with the statements “Yes” and “No” and excluded “don’t know/not sure/depends” statements. The stigma variable was then categorized into “No” if the respondents answered “No” to zero to two statements and “Yes” if the respondents answered “Yes” to three or four statements.

HIV-related knowledge was defined as the respondents’ knowledge about HIV. This variable was constructed from six variables, namely, “Reduce risk of getting HIV: Always use condoms during sex,” “Reduce risk of getting HIV: Have one sex partner only, who has no other partners”, “Can get HIV by sharing food with a person who has AIDS”, “HIV transmitted during pregnancy”, “HIV transmitted during delivery”, and “HIV transmitted by breastfeeding.” Each variable was then recorded as “Yes” and “No” to determine the level of HIV knowledge [34]. The response of “do not know” was excluded. The knowledge variable was then categorized into “No Knowledge” if the respondents answered “Yes” to two variables, “Some Knowledge” if the respondent answered “Yes” to three or four variables, and “More Knowledge” if the respondents answered “Yes” to five or six variables.

HIV-related information was defined as the information about HIV/AIDS that the respondent receives. We constructed four questions that ask what the respondents received in terms of information about HIV/AIDS. The variables were sources for AIDS knowledge: “internet”, “newspaper/magazine”, “health professional”, and “school/teacher” [34]. Each variable was recoded with the statements “Yes” and “No” to obtain a consistent response for the sources of information on HIV/AIDS. All of the variables were compiled to obtain the new “information” variable. This variable was then categorized into three groups, namely, “No Information” if the respondent answered “No” for all questions, “Some Information” if the respondent answered “Yes” to one question, and “More Information” if the respondent answered “Yes” to two to four questions.

The strengthening the reporting of observational studies in epidemiology, or STROBE, guidelines were followed in the analysis and reporting of these findings [36].

### 2.4. Data Analysis

We analyzed the data using STATA/MP version 16.1. Chi-square and binary logistic regression were performed to analyze the data and interpret the study results. We used an adjusted odds ratio (AOR) with a 95% confidence interval (CI) and a significance level of *p* < 0.05.

### 2.5. Ethical Consideration

The 2017 Indonesian Demographic and Health Survey was approved under the Institutional Review Board (IRB) Findings Form ICF IRB FWA00000845. DHS obtained written informed consent from each individual. The information about the ethical review is available on the website (https://dhsprogram.com/Methodology/Protecting-the-Privacy-of-DHS-Survey-Respondents.cfm (accessed on 8 November 2021)).

## 3. Results

From the total of 2118 respondents in eastern Indonesia, the majority of respondents were distributed in Sulawesi (64.54%). More than 70% of the respondents did not engage in HIV testing at their local PHC. In this study, more than 40% of respondents were aged 35–49 years; the majority, 79.93%, were women; 23.09% were in the richest wealth category; 49.39% had attained higher education; and 51.94% lived in urban areas. It also became known that 92.35% of respondents were working; 86.83% received earnings; 67.75% did not engage in stigmatizing behavior related to HIV; and 71.39% of respondents had more knowledge of HIV, while 42.02% responded that they had some information of HIV (Table 1).

**Figure 2:**
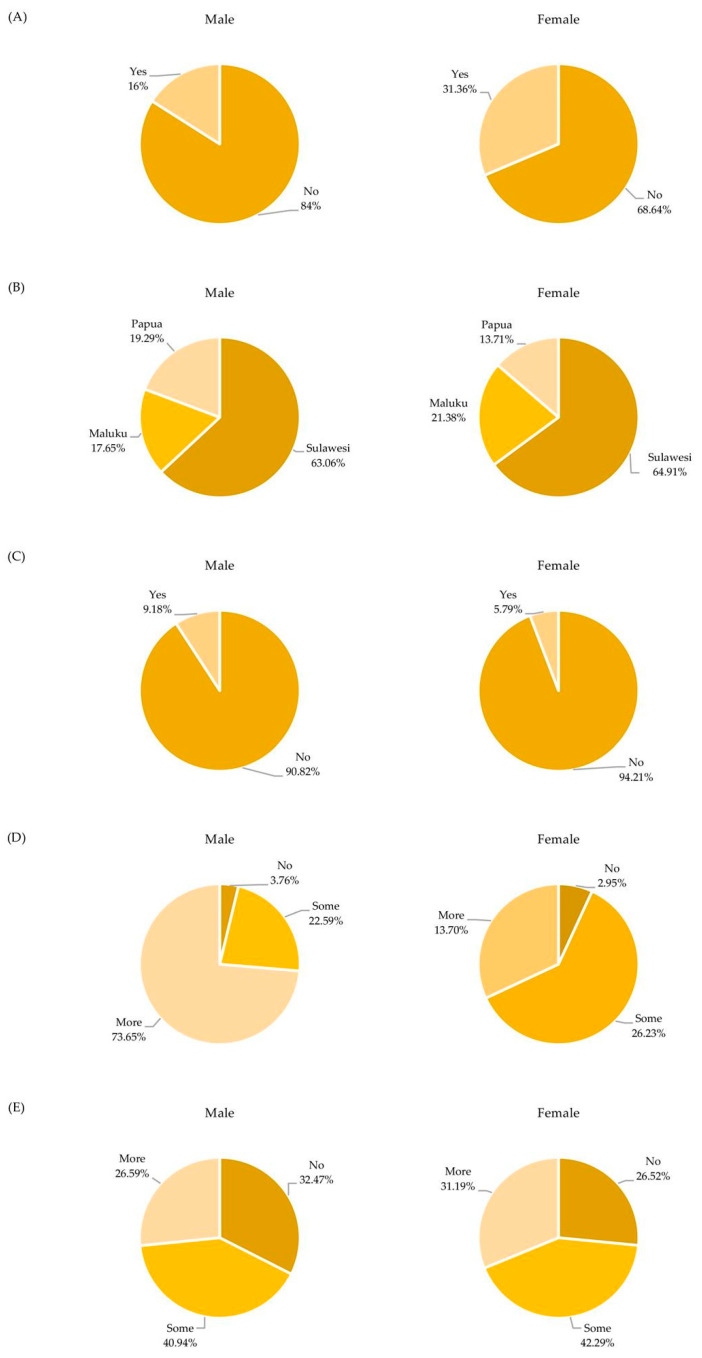
Pie Charts of the Distribution of Data for Males and Females in Eastern Indonesia for (**A**) HIV Testing, (**B**) Regions, (**C**) Stigma, (**D**) HIV-Related Knowledge, and (**E**) HIV-Related Information.

**Figure 3:**
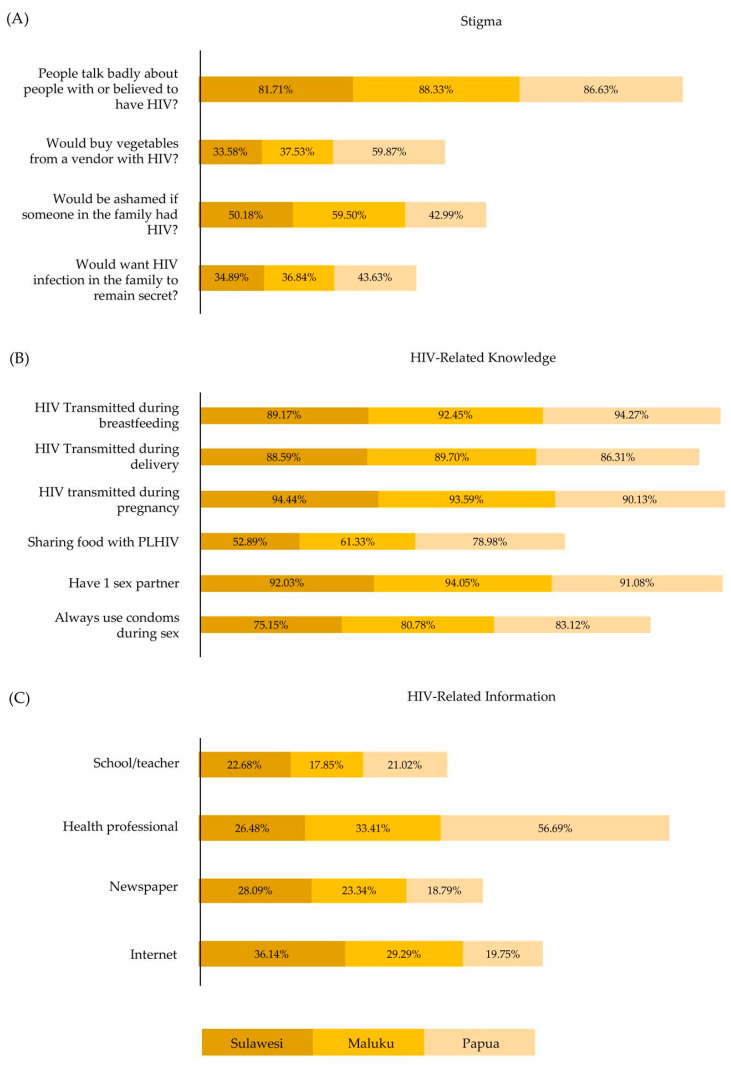
Bar Charts of the Distribution of Data for Eastern Indonesia (Sulawesi, Maluku, and Papua) for (**A**) Stigma, (**B**) HIV-Related Knowledge, and (**C**) HIV-Related Information.

Figure 2 summarizes the distribution of males and females in eastern Indonesia according to HIV testing, regions, stigma, and HIV-related knowledge and information. Figure 3 presents the description of stigma, HIV-related knowledge, and HIV-related information for eastern Indonesia. More than 80% of the people in eastern Indonesia talked badly about PLHIV. Regarding HIV-related knowledge, education about sharing food with PLHIV should be promoted. Less than 80% of people believe that HIV/AIDS can be transmitted by sharing food with PLHIV. However, less than 50% of people in eastern Indonesia received information from schools, health professionals, newspapers, and the internet. This should be promoted by the government.

The regional distribution in eastern Indonesia is shown in Table 2. We present data on three provinces from eastern Indonesia. Most of the HIV testing was conducted in Papua, and most of the respondents in eastern Indonesia were aged 35–49 years. Most of the respondents were female and respondents who lived in Sulawesi were in the richest wealth index. This was conversely so in Maluku and Papua. Most of the respondents had a high education level. The respondents in Papua were mostly living in rural areas, and this was conversely found in Sulawesi and Papua. Most of the respondents in eastern Indonesia were working and making earnings. Stigmatized behavior was mostly not presented by the respondents in all regions. Furthermore, most of the respondents have more knowledge and some information about HIV.

Table 3 presents the bivariate and multivariate analyses. On the basis of the bivariate analysis, we found that region, age, gender, wealth index, stigma, and information have a significant correlation with HIV testing in PHCs in eastern Indonesia. We then conducted the multivariate analysis. The respondents in Papua were 1.45 times more likely to have an HIV test in a PHC (AOR: 1.45; 95% CI: 1.09–1.91) than the respondents in Sulawesi and Maluku. Female respondents were 2.37 times more likely to have an HIV test in a PHC (AOR: 2.37; 95% CI: 1.75–3.12) than males. When assessing the wealth index, the respondents with the richest level were found to be 0.61 times less likely to have an HIV test in a PHC (AOR: 0.61; 95% CI: 0.42–0.89) compared to the other wealth index levels. Furthermore, the respondents who engaged in and/or experienced stigmatization were 0.65 times less likely to have an HIV test in a PHC (AOR: 0.65; 95% CI: 0.42–1.02) than the respondents who did not. The respondents with more information about HIV were 1.88 times more likely to have an HIV test in a PHC (AOR: 1.88; 95% CI: 1.41–2.51) compared to those with other levels of information.

## 4. Discussion

HIV/AIDS management can involve utilizing the optimization of HIV testing and screening. Data from the Indonesian Ministry of Health indicates that there are suboptimal levels of HIV testing. In most Asian countries, HIV/AIDS is concentrated among at-risk populations [9]. Late HIV diagnosis increases the risk of HIV transmission to others, as well as the chance of eventual illness and mortality and expensive health treatment [37]. Previous studies conducted in seven countries in the Asia–Pacific region, including Indonesia, revealed that the barriers to HIV testing were as follows: young age, engaged in sex work, transgender, injected drugs, perceived deficiency in health status, lack of insurance, fear of violation of confidentially, and self-referred for HIV testing [17,18,38,39]. However, several components were found to affect the prevalence of HIV testing in this study, including gender, wealth, region, stigma, and information related to HIV.

In this study, we found that female respondents are more likely to undergo an HIV test at a PHC. The low level of health-seeking behaviors in males to pursue HIV testing services in other relevant clinical settings could explain why fewer males were accessing HIV testing and treatment [40]. It has been reported that the most significant contributor to the higher percentage of female than male participants taking HIV tests at the PHCs is because women were undergoing HIV testing during pregnancy [41]. The global screening of pregnant women is essential to prevent the mother-to-child transmission (MTCT) of HIV through provider-initiated routine testing and antiretroviral therapy (ART) in antenatal clinics [10,42,43]. In line with the previous studies, the results reported that women had a higher percentage of undergoing HIV tests in the last 12 months in five out of sixteen countries [40,44]. However, many women have still not yet been tested for HIV/AIDS [41]. It was noted that not all accepted the screening. Of the 100% target of pregnant women in PHCs, 30% disagreed with taking the test [45]. Some of the reasons included being unable to make an informed decision, having no right to secrecy and privacy, facing continuing discrimination, and having no access to prompt HIV testing. Indonesia, therefore, needs to dramatically increase its rate of HIV screening and ART [46].

Meanwhile, the wealthiest respondents were less likely to engage with HIV tests performed at a PHC. This is related to the previous studies where wealth is shown to be a predictor of HIV testing. HIV testing rates tend to rise in lockstep with wealth [40]. There is an association between family income and the utilization of health services. Respondents who have a low family income and who use health services total 28%. The level of public awareness encourages them to take advantage of the subsidies provided by the government at no cost as part of the national health insurance program. A high family income will influence the decision-making process when it comes to seeking better health services to improve their health status. The community knows that to obtain quality health services, they have to pay. Therefore, people who have a high family income prefer to visit the health services they think are better, such as hospitals or practicing doctors further away from the PHC [10].

HIV testing rates at the PHCs in eastern Indonesia was found to be 28.28%. In this study, the respondents in Papua are more likely to have an HIV test. On the basis of previous studies, we recommend a local, culture-based approach for disseminating the information about HIV testing in regions similar to eastern Indonesia [8,21]. By using local language, the information will be well delivered in the society. A culture-based approach and the closeness of the health workers to the community are the right ways to go about reaching the population. This approach showed great improvement and resulted in a high interest of Papua’s community in the HIV testing at the PHCs.

The respondents who experienced stigmatization were less like to take an HIV test at a PHC. In this study, stigma is shown by their HIV diagnosis remaining secret, feeling ashamed if someone in the family has HIV, not buying vegetables from vendors with HIV, and talking badly about people with or believed to have HIV. Contextual variables, such as the community-level stigma toward HIV-positive persons, may potentially influence the validity of self-reported HIV tests [40]. A previous study reported that one reason for the lower uptake of the HIV testing service in most low- and middle-income countries is HIV stigma. HIV stigma is linked to the decreased use of HIV testing services, non-disclosure, and delayed access to comprehensive health treatment, leading to higher transmission rates [47].

The last contributor to performing HIV tests at PHCs was more information about HIV. Access to information and peer support plays a significant role as a predictor of HIV testing among the participants. This result is reliable when compared to the findings of the previous research in the field, such as the access to high-quality information about HIV testing services resulting in an increase in the proportion of sex workers seeking HIV testing. These findings are also in line with the previous research that identified a relationship between the quality of the media information on HIV and the participants’ willingness to undergo HIV testing in a PHC [9]. The use of adequate information will help to increase the coverage of HIV testing and PHC optimization.

### 4.1. Limitations

This study was confined to eastern Indonesia and in PHC settings. The findings may not be generalizable to other regions. This is because eastern Indonesia is different in cultural, socio-economic, and level of education factors. Thus, the data and information obtained in this study were only specific to eastern Indonesia and might affect the generalizability of the data. However, the government’s programs in the coverage of HIV testing in Indonesia as a whole have similarities.

### 4.2. Recommendation

The national coverage in Indonesia will give general information to provide specific policies related to HIV testing and treatment. Meanwhile, the local, culture-based approach in each region in Indonesia can be a consideration to improve the reach of HIV testing coverage. More funding in HIV programs is needed to improve both prevention and treatment, and to increase the number of diagnoses made at an earlier stage. Furthermore, increasing equity, distributing information regarding HIV/AIDS through social media, and creating accessible HIV testing in rural areas are vital for developing appropriate interventions.

## 5. Conclusions

This study analyzed the current information regarding the optimizing of HIV testing in eastern Indonesia from PHC settings as the first-level health facility in Indonesia. The study revealed that HIV testing in eastern Indonesia was significantly associated with many factors, such as gender, wealth, region, stigma, and information related to HIV. Optimizing PHCs as a first-level health facility that are close to the local community for HIV testing should be considered. The dissemination of information about HIV/AIDS on reducing stigma needs to be enhanced. As Indonesia consists of various cultures, a local, culture-based approach can be implemented to reach the community for HIV testing and screening. With this study, the government may determine further policies regarding the screening and control of HIV infection, especially in eastern Indonesia.

## Figures and Tables

**Table 1:** Respondents’ Characteristics (*n* = 2118).

Variable	*n*	%
HIV Test		
No	1519	71.72
Yes	599	28.28
Region		
Sulawesi	1367	64.54
Maluku	437	20.63
Papua	314	14.83
Age		
50–54 years	41	1.94
35–49 years	943	44.52
25–34 years	752	35.51
15–24 years	382	18.04
Gender		
Male	425	20.07
Female	1693	79.93
Wealth Index		
Poorest	382	18.04
Poorer	406	19.17
Middle	377	17.8
Richer	464	21.91
Richest	489	23.09
Education		
Higher education	1046	49.39
Secondary education	930	43.91
Primary education	136	6.42
No education	6	0.28
Residence		
Urban	1100	51.94
Rural	1018	48.06
Respondents working		
No	162	7.65
Yes	1956	92.35
Respondents earning		
No	279	13.17
Yes	1839	86.83
Stigma		
No	1435	67.75
Yes	683	32.25
Knowledge		
No	66	3.12
Some	540	25.5
More	1512	71.39
Information		
No	587	27.71
Some	890	42.02
More	641	30.26

**Table 2:** Regional Distribution in Eastern Indonesia (n = 2118).

Variable	Eastern Indonesia					
	Sulawesi		Maluku		Papua	
	*n*	%	*n*	%	*n*	%
HIV Test						
No	1001	73.23	314	71.85	204	64.97
Yes	366	26.77	123	28.15	110	35.03
Age						
50–54 years	32	2.34	3	0.69	6	1.91
35–49 years	606	44.33	200	45.77	137	43.63
25–34 years	468	34.24	160	36.61	124	39.49
15–24 years	261	19.09	74	16.93	47	14.94
Gender						
Male	268	19.60	75	17.16	82	26.11
Female	1099	80.40	362	82.84	232	73.89
Wealth Index						
Poorest	180	13.17	117	26.77	85	27.07
Poorer	245	17.92	95	21.74	66	21.02
Middle	222	16.24	90	20.59	65	20.70
Richer	309	22.60	91	20.82	64	20.38
Richest	411	30.07	44	10.07	34	10.83
Education						
High education	699	51.13	220	50.34	127	40.45
Secondary education	573	41.92	197	45.08	160	50.96
Primary education	93	6.80	19	4.35	24	7.64
No education	2	0.15	1	0.23	3	0.96
Residence						
Urban	716	52.38	257	58.81	127	40.45
Rural	651	47.62	180	41.19	187	59.55
Respondents working						
No	114	8.34	21	4.81	27	8.60
Yes	1253	91.66	416	95.19	287	91.40
Respondents earning						
No	150	10.97	67	15.33	62	19.75
Yes	1217	89.03	370	84.67	252	80.25
Stigma						
No	968	70.81	287	65.68	180	57.32
Yes	399	29.19	150	34.32	134	42.68
Knowledge						
No	46	3.37	11	2.52	9	2.87
Some	338	24.73	108	24.71	94	29.94
More	983	71.91	318	72.77	211	67.20
Information						
No	394	28.82	126	28.83	67	21.34
Some	544	39.80	193	44.16	153	48.73
More	429	31.38	118	27.00	94	29.94

**Table 3:** Bivariate and Multivariate Analysis of Optimization of HIV Testing in Eastern Indonesia.

Variable	HIV Test				X2	AOR	95% CI	
	No		Yes					
	*n*	%	*n*	%			Lower	Upper
Region								
Sulawesi	1001	47.26	366	17.28	8.58 **	Ref.		
Maluku	314	14.83	123	5.81		1.004	0.77	1.29
Papua	204	9.63	110	5.19		1.45 ***	1.09	1.91
Age								
50–54 years	36	1.7	5	0.24	10.80 **	Ref.		
35–49 years	692	32.67	251	11.85		1.29	0.48	3.46
25–34 years	535	25.26	217	10.25		1.24	0.45	3.35
15–24 years	256	12.09	126	5.95		1.34	0.48	3.71
Gender								
Male	357	16.86	68	3.21	39.53 ***	Ref.		
Female	1162	54.86	531	25.07		2.37 ***	1.75	3.12
Wealth Index								
Poorest	266	12.56	116	5.48	10.11 **	Ref.		
Poorer	289	13.64	117	5.52		0.92	0.67	1.28
Middle	263	12.42	114	5.38		0.95	0.68	1.32
Richer	323	15.25	141	6.66		0.93	0.66	1.3
Richest	378	17.85	111	5.24		0.61 **	0.42	0.89
Education								
Higher education	734	34.66	312	14.73	4.36	Ref.		
Secondary education	685	32.34	245	11.57		0.98	0.78	1.23
Primary education	97	4.58	39	1.84		1.17	0.75	1.82
No education	3	0.14	3	0.14		2.71	0.51	14.38
Residence								
Urban	806	38.05	294	13.88	2.72 *	Ref.		
Rural	713	33.66	305	14.4		1.09	0.88	1.35
Respondents working								
No	113	5.34	49	2.31	0.33	Ref.		
Yes	1406	66.38	550	25.97		1.15	0.79	1.67
Respondents earning								
No	206	9.73	73	3.45	0.7	Ref.		
Yes	1313	61.99	526	24.83		1.30 *	0.96	1.78
Stigma								
No	1023	48.3	412	19.45	4.44 **	Ref.		
Yes	496	5.15	187	1.32		0.65 **	0.42	1.02
HIV-related Knowledge								
No	47	23.42	19	8.83	5.14 *	Ref.		
Some	367	17.33	173	8.17		1.18	0.66	2.1
More	1105	52.17	407	19.22		0.94	0.53	1.64
HIV-related Information								
No	451	21.29	136	6.42	20.21 ***	Ref.		
Some	648	30.59	242	11.43		1.27 *	0.98	1.64
More	420	19.83	221	10.43		1.88 ***	1.41	2.51

*** *p* < 0.01, ** *p* < 0.05, * *p* < 0.1, AOR: Adjusted Odd Ratio, CI: Confident Interval, X2: Chi-square.

## Data Availability

The data presented in this study are available on request from the corresponding author.
